# Supplementary material for: Tolerability and pharmacokinetic evaluation of inhaled dry powder hydroxychloroquine in healthy volunteers
Source: PLoS One. 2022 Aug 5;17(8):e0272034. doi: 10.1371/journal.pone.0272034 (PMC9355221; doi:10.1371/journal.pone.0272034)
Supplement: Attachment — Submitted filename: Respons to reviewer.docx [file pone.0272034.s002.docx]

# Response to reviewers

We would like to thank the editor and reviewers for their time, critical view and suggestions to improve our manuscript.

The following additional requirements were implemented:

1. We applied the PLOS ONE style requirements
2. We provided the full name of the Institutional Review board [line 116].
3. A and C: Additional information about the participant recruitment method, recruitment date range [line 109-112] and table with demographic details of the participants [table 2] are added.
   B: We made a statement in the discussion section whether our sample can be considered representative of a larger population [ see comment on reviewer 2]
4. We included the tables with separate captions in the manuscript and removed them as separate files.
5. The figure was given a separate caption in the manuscript.
6. We did not add new supporting information files with this resubmission
7. We updated the Competing Interests Statement and the Funding Statement as mentioned in cover letter and in the manuscript.
8. We declared the use of a patented inhaler called the Cyclops with number WO2015/187025 in the cover letter.
9. We reviewed and completed the reference list. The references to the unpublished data of Dayton et al were replaced [reference 37, 38, 39], since their results were not directly available to us and only published after personal communication in the article of Kavanagh et al [reference 34]. The references belonging to the retracted article of Mehra et al [56] and the retraction notice by Watson et al [57] are removed. New references are added as mentioned in the response to the reviewers.

Below is our response to each point raised by the reviewers. Line numbers correspond with the lines in the ‘revised mancuscript track and changes’

## Reviewer 1:

**This is a well written concise manuscript describing a small safety study in healthy volunteers to explore the use of inhaled dry powder hydroxychloroquine to treat COVID 19 in the future. The authors correctly point out that this study does not provide proof of principle that the dose and administration used here produces the drug levels in alveolar tissue that would definitely inhibit viral invasion.**

**The authors could add some sentences to discuss whether a separate study to attempt to measure alveolar levels in healthy subjects should be done.**

**I think the authors should comment on whether this inhaler would produce alveolar deposition or whether it is limited to larger airways. A comment on ANY DEPOSITION STUDIES would help**

Thank you for these suggestions.

It is correct that this study does not provide proof of principle that the dose and administration used here produces alveolar tissue concentrations that inhibit viral invasion or replication. The limitation of the study is that we only measured hydroxychloroquine systemically – in blood samples – but ideally this should be measured in lung tissue, BAL, and epithelial lining fluid as well. It would be of great interest to know what concentration can be achieved after inhalation and compare this with oral dosing. We added this limitation to the discussion section [line 290-294].

We made the expectation that there will be alveolar deposition with use of the Cyclops more clear in the introduction section by adding information about the particle size produced by the inhaler [line 92-97].

## Reviewer 2:

**The major problem with the study is the sample size which is 12. However, due to the fact that the authors use only a t-test, this is potentially acceptable.**

**The authors should add a sample size estimation based on reasonable assumptions for the effect size to demonstrate that for the studied problem this small sample size is tolerable. In addition, please emphasize in the discussion this issue.**

We agree on the comment of the small sample size. It is difficult to make a specific calculation of the number of subjects needed in this pilot study. Low numbers have provided good results in other dry powder inhalation studies of antibiotics [41-44], so we think that 12 participants are sufficient to make a good impression of safety and tolerability. This is supported specifically for HCQ by the phase 1 study of aerosolized HCQ in 31 healthy individuals and the phase 2 with aerosolized HCQ in patients with asthma [34]. We added a note of this in the discussion section and provided new supporting references accordingly [line 271-287].

## Reviewer 3:

**This manuscript is a well written paper with clear study design and conclusions. However, there are some minor points to be addressed.

1. Please describe more details for the study design including dosing sequence, wash-out period, food (fasting or fed), restrictions.**

We provided extra information in the manuscript regarding the wash-out period of at least 4 days in the methods section. As mentioned in the manuscript the dosing sequence consisted of single doses, starting with a dose of 5 mg, ascending tot 10 and finally 20mg [line 112-114]. Feeding state and restrictions are important point to address in drug research, but since the drug in this study is deposited and resorbed from the lungs it holds less relevance. Therefore we did not document feeding state or other restrictions in the manuscript.

**2. Please describe or discuss the rationale for the time points of FEV1 assessments and ECG assessment.**

The timepoints for spirometry were chosen shortly after inhalation, because bronchoconstriction as a side effect is expected to occur shortly after inhalation due to direct irritating effects. This has been shown in other studies with inhaled antimicrobials, for example in the article of Hoppentocht et al where drop in FEV1 > 10% is observed within 95 minutes after inhalation of dry powder tobramycin [41][line 135-138].

The risk of QTc prolongation increases with higher systemic HCQ concentrations. We added ECG assessment as a safety parameter, although (high) systemic concentrations were not to expected after local administration in a dose that is just a fraction of the oral dose [line 260-268]. Since there was no data available about the absorption rate of HCQ from the lungs we based our time point on the time that HCQ reaches a maximum concentration after oral dosing, which is within 2-4.5 hours. For logistical reasons we planned it at the end of the study day which was 3,5 hours after inhalation [line 295-296].

**3. It seems to be better to present the adverse events according to dose in Table 3.**

Thank you for this suggestion. We updated the table en presented adverse events according to dose and in absolute numbers instead of percentages in table 3.

**4. This study aimed to evaluate the tolerability and PK after a single dose. However, in clinical setting, multiple doses will be needed for the treatment of COVID-19. Please add the limitation of this single dose study in Discussion.**

We agree that the single dose is a limitation in our study since in COVID-19 treatment multiple doses will be needed. We added this to the limitations in the discussion section [lines 275-277].

## Reviewer 4:

**This paper describes a simple but useful trial to test, in healthy volunteers, the pulmonary safety and tolerability of an inhaled, dry-powder formulation of hydroxychloroquine. It deserves rapid publication, because it's of topical interest as regards the treatment of COVID-19. The authors should add comments or discussion about the following weaknesses of the trial:**

1. **The trial is open-label and has no placebo control, so no statistical hypothesis testing is justified. The lack of placebo weakens the assessment of tolerability and safety, including the main pulmonary outcome measure. (FEV1).**

The study indeed is open label without placebo control. We believe this limitation is more important when one does find safety or tolerability problems and perspective is needed, but not so much when problems found are minor anyway.

2. **The trial did not include any measures of pulmonary inflammation (such as exhaled NO) or gas permeability (eg CO transfer factor)**

We agree that pulmonary inflammation as such was not measured, nor potential proxies such as FeNO or KCO. This will be interesting to consider in a next, phase 1b study in which we will aim for measuring direct local concentrations in the airways and BAL.

3. **In line 235, the authors refer to the rapid "elimination" of hydroxychloroquine: the word "distribution" would be more accurate.**

Indeed the word distribution is more accurate, we replaced the word elimination.

4. **There are a few typographical errors to be corrected. Also, the phrase "allow for" has been used throughout: the "for" is incorrect in this context, and "allow" should be used on its own.**

We deleted the word ‘for’ in this context and checked the manuscript again for typographical errors.

5. **Complete data are not available**

All relevant data are within the paper. The complete data set can be requested from the authors.

## Reviewer 5:

**The article is well written and can have an impact in clinical practice. The major disadvantage is related to the time when the blood samples were taken, too late to measure the plasma concentration of the drug.**

We agree this is an disadvantage and added this as a limitation of the study in the discussion section [line 293-299]. Timepoints for drawing the blood samples were based on the rate of absorption after oral administration of HCQ, since data after pulmonary absorption were lacking at time of writing of the study protocol. Based on results and current knowledge blood samples should be taken at earlier time points as well in future studies.

**Table 3 mention "respiratory infection", but this term does not appear in the manuscript. How did you evaluate this side effect?**

In table 3 we changed ‘respiratory infection/sore throat’ to ‘sore throat’. Participants complaining of sore threat or other upper respiratory tract infection symptoms were provided a swab for respiratory viruses of which one tested positive for SARS-CoV-2, as mentioned in the manuscript [line 191-194].

**In my opinion, "inhaled dry powder HCQ is safe" is too enthusiastic statement, because you have followed a small number of patients for a short time after single increasing doses of 5, 10 and 20 mg. I think you need to mention this within the limits of the study.**

We agree on this comment and added this as a limitation in the discussion section. See comments made by reviewer 2 and reviewer 3. In addition we nuanced the conclusion [line 45-47 and line 334-335].
