# Supplementary material for: Tolerability and pharmacokinetic evaluation of inhaled dry powder hydroxychloroquine in healthy volunteers
Source: PLoS One. 2022 Aug 5;17(8):e0272034. doi: 10.1371/journal.pone.0272034 (PMC9355221; doi:10.1371/journal.pone.0272034)
Supplement: Attachment — Submitted filename: Respons to reviewer 02-01-2022.docx [file pone.0272034.s003.docx]

# Response to reviewers

We would like to thank the editor and reviewers for their time, critical view and suggestions to improve our manuscript.

The following additional requirements were implemented based on the suggestion from reviewer 6. The study design was clear, and the manuscript was well written in general. I would recommend authors to briefly mention the method of development of HCQ dry powder formulation and it’s aerosolization behavior in vitro as this study couldn’t test the lung concentrations of drug before acceptance for publication

In response to reviewer 6 we added additional information to the methods section, under 'study drug', about the development of the HCQ dry powder formulation and it’s behavior in vitro. [line 107-115].

The HCQ Cyclops was produced by PureIMS B.V. (Roden, the Netherlands). For this, hydroxychloroquine sulphate was comicronized by air jet milling with 4% L-leucine to improve formulation dispersion. Each Cyclops contained a nominal dose of 5 mg or 10 mg HCQ; the 20 mg dose was administered as 2 successive inhalations with 10 mg of HCQ. The doses were comparable with known doses of inhaled nebulized HCQ and thus expected to be safe (34, 36). Furthermore, to enhance dose emission from the inhaler, 5 mg of coarse lactose was added to the dose compartment. At 4 kPa, 85% of the nominal dose was emitted from Cyclops with a fine particle fraction < 5 µm of 74% (i.e. 63% of the nominal dose), as determined by laser diffraction analysis.
