# Supplementary material for: Tolerability and pharmacokinetic evaluation of inhaled dry powder hydroxychloroquine in healthy volunteers
Source: PLoS One. 2022 Aug 5;17(8):e0272034. doi: 10.1371/journal.pone.0272034 (PMC9355221; doi:10.1371/journal.pone.0272034)
Supplement: Attachment — Submitted filename: Respons to Reviewer iHCQ.docx [file pone.0272034.s004.docx]

# Response to Reviewers

We would like to thank the editor and reviewer for their time, critical view and suggestions to improve our manuscript.

1. **The topic is misleading on 'Pharmacokinetics Evaluation. I have not seen any in the manuscript related to that.**

The pharmacokinetic evaluation consisted of measuring serum HCQ concentrations predose, 30 minutes, 2 and 3,5 hours after inhalation. Serum HCQ concentrations are reported in the manuscript. Please refer to line 202-206

“In all participants, the serum HCQ concentrations sampled predose, 30 minutes, 2 and 3.5 hours, were below the detection level of 10 µg/L, irrespective of dose. The mean delivered dose was 3.16 mg (63%) after administration of 5 mg HCQ, 6.95 mg (70%) after administration of 10 mg HCQ and 14.86 mg (75%) after administration of 20 mg HCQ. Based on the recorded inspiratory flow parameters, all participants correctly performed the inhalation maneuvers.”

2. **The statement in the first two paragraphs of the introduction section is obsolete. The authors need to update at least as of December 31, 2021.**

Thank you, we agree. Unfortunately, the manuscript has been under review for two very long periods at the journal. The information is now updated.

3. **The authors must mention many available treatment modalities, including the recent EUA COVID pill.**

Thank you for this suggestion. We added a paragraph with a short summary of current treatment advice according to the WHO living guideline accessed June 26^th^, 2022.

4. **Why was the oral HCQ not advised by FDA and EMA? The authors need to refer to the RECOVERY Trial and the prospective European DISCOVERY and WHO SOLIDARITY trials. Those details would enhance the manuscript.**

Yes, it is important to know that oral HCQ was not adviced because of a lack of effect on mortality. In line 78-81 we now refer to this decision and the mentioned trials.

5. **The statistical analytic plans are critically important in ensuring appropriate reporting of clinical trials. The significance reported using the methods seems irrelevant due to the small sample size and the authors' need to consider using the exact tests. The main focus is the safety and tolerability, not the statistical significance of the measures collected.**

We would like to thank the reviewer for this advice. Since the main focus is indeed the safety and tolerability and the sample size is small we decided not to mention the statistical analysis anymore and only use descriptive statistics. Therefore we changed the text in the data management and statistics section and removed the p-values from the tables.

6. **There is no uniformity in using the terminology. In some places in the manuscript, the authors use 'volunteer' and state 'participant' in many areas.**

Thank you for this suggestion to make the manuscript more uniform. As volunteers participate in the study they can also be referred to as participants. The term healthy volunteer is now only used in the methods sections, in the rest of the manuscript we use the term participant.

7. **The second paragraph in the results section (lines 165- 184) should be converted into a table by listing all event names with a number of participants. Then, delete the entire text from that section.**

Thank you, we agree that a table gives the best overview of the events, they are mentioned in table 3. To our opinion, the text adds more detailed information to this. Therefore we decided not to remove the text.

8. **Not clear, the authors screened 12 participants, and all are eligible and enrolled in the study? If it is more than that, it has to be reported.**

All participants screened were eligible and were enrolled in the study. This has now been made more clear in the text.

9. **The statements in the discussion section (Lines 217-227) should highlight the importance of the study and focus on elaborating the contents based on the data with supporting evidence (any prior cited research). The statistical values reported in that paragraph in the lines 229-240 should be deleted.**

Thank you, we tried to make the importance of the study more clear in the first paragraph of the discussion and added citations to previous mentioned research in the first and second paragraph as well.

10. **The authors described why the timing was chosen (Lines 268-272) for blood collection. These statements should move to the methods section.**

We moved the statement to the methods section as suggested.

11. **There is not enough in the discussion to claim this study is warranted. A strong justification is still missing: why is the clinical research warranted? How would the proposed method help in the clinical arena?**

There is not a good explanation why hydroxychloroquine lacks a clinical effect in the treatment of COVID-19 despite the promising results of in vitro studies and the assumed mechanism of action. We tried to state this more clear in the first section of the discussion section, addressing that we hypothesize that high enough concentrations are not reached in the lung where needed when dosed systemically, because of limitations of systemic toxicity, but might be reached by inhaled dosing.

**12. Line 198: Table 1: Check the spelling for 'Cough.' It says 'Couch.'**

We corrected the spelling

12. **Line 203: Table 3: Last column 'Percentage of patients' is meaningless- should be excluded. Add the percentage under each dose column N (%).**

Thank you. We excluded the last column and added the percentages under the remaining column as suggested.

**14. Line 205: The change in value for 10 mg HCQ from baseline to 95 minutes post inhalation is reported as 0.21. All the other values are less than zero. The authors need to double-check the calculation of this declared value.**

We checked this value and the value of +0.21 % is correctly documented.
